# Reduced muscle strength (dynapenia) in women with obesity confers a greater risk of falls and fractures in the UK Biobank

**DOI:** 10.1002/oby.23609

**Published:** 2022-12-11

**Authors:** Lisa Dowling, Daniel J. Cuthbertson, Jennifer S. Walsh

**Affiliations:** ^1^ Oncology and Metabolism Medical School, The University of Sheffield Sheffield UK; ^2^ Department of Cardiovascular and Metabolic Medicine, Institute of Life Course and Medical Sciences The University of Liverpool Liverpool UK; ^3^ Liverpool University Hospitals NHS Foundation Trust Liverpool UK

## Abstract

**Objective:**

This study aimed to determine the independent effects of obesity and dynapenia on falls risk, areal bone mineral density, and fracture risk (lower extremity or all other fractures).

**Methods:**

A total of 16,147 women (aged 60‐82 years) from the UK Biobank were categorized by handgrip strength (HGS; dynapenia status: HGS ≤ 21 kg) and body weight (BMI: normal weight, overweight, or obesity). Multiple logistic regression models examined the association among dynapenia and obesity and self‐reported falls (previous 12 months), lower extremity fractures, and all other fractures (previous 5 years).

**Results:**

A total of 3793/16,147 women fell, and 1413/15,570 (9.1%) eligible women experienced fall‐related fractures. Obesity (odds ratio [OR] 1.25; 95% CI: 1.12‐1.38) and dynapenia (OR 0.87; 95% CI: 0.77‐0.98) were both independently associated with greater lower extremity fracture risk, independently of areal bone mineral density. However, considering all other fracture sites, obesity conferred protection (OR 0.77; 95% CI: 0.61‐0.96), except in those with low HGS, who had an equivalent fracture risk to those with normal weight (OR 1.06; 95% CI: 0.82‐1.38).

**Conclusions:**

Dynapenia further increases the increased risk of leg and ankle fractures in obesity and counteracts the protective effects of obesity on fracture risk at all other sites (wrist, arm, hip, spine, other bones).

**Study Importance**

**What is already known?**

- People with obesity have a greater risk of fractures at the ankle, lower leg, and proximal humerus but a lower risk of other fractures; however, the mechanisms are unclear.
- Low muscle strength (dynapenia) is associated with a greater risk of fracture.
- A recent systematic review found no cumulative effect of obesity and dynapenia on nonvertebral fractures; however, this group of fractures has heterogeneous risk factors and mechanisms, especially in obesity.

**What does this study add?**

- Dynapenia, irrespective of bone mineral density, further increases the risk of lower extremity (ankle and leg) fractures in women with obesity.
- Dynapenia counteracts the lower risk of all other fractures in women with obesity.

**How might these results change the direction of research or the focus of clinical practice?**

- These findings highlight the independent risks of these phenotypes (obesity and dynapenia) and contribute to the understanding of the site‐specific fracture risk in this group.
- Clinically, these results highlight the importance of increasing physical activity and exercise in weight management programs.

## INTRODUCTION

Obesity is associated with a greater risk of fracture at the ankle, lower leg, and proximal humerus [1, 2, 3] but a lower fracture risk at the wrist, hip, and spine [2, 4, 5]. A more injurious fall type may explain this site‐specific fracture risk given the knowledge that obesity is associated with greater areal bone mineral density (BMD) and stronger bone structure, such as with denser cortices and increased trabecular number and thickness [5, 6, 7]. A greater understanding of fracture risk in a population with a growing prevalence of obesity could reduce the economic burden of fracture through targeted prevention strategies; the medical and social cost of fragility fractures alone is estimated at £4.4 billion per year in the UK [8].

The measurement of lean mass in obesity is problematic owing to scaling with body size [9], and it is also becoming increasingly apparent that lean mass may not be the best surrogate of muscle mass [10]. Dynapenia, or low muscle strength, has been recommended in recent reports and definitions of sarcopenia [11, 12]. As muscle strength reduces at a greater rate than muscle mass with age, and as this reduction is only partially explained by muscle mass, the concept of dynapenia has been identified as a distinct condition [13]. Dynapenic abdominal obesity refers to the combination of low muscle strength and obesity, which is estimated to affect 3.6% to 23.4% of older adults [14, 15, 16, 17, 18]. Prevalence is higher in smaller studies [17, 18] and is approaching ~10% in large and population‐based studies [14, 15, 16]. There is a bidirectional relationship between obesity and dynapenia: obesity may exacerbate dynapenia (e.g., through secretion of proinflammatory cytokines), whereas dynapenia may exacerbate weight gain with an impaired ability to undertake physical activity [19].

Independently, dynapenia and obesity are associated with a greater risk of falls [20, 21, 22], with reports suggesting a synergistic effect on falls risk [17, 18, 23]. Dynapenia is associated with a greater risk of all fractures [24], whereas obesity is associated with site‐specific fracture risk [1, 2, 3, 4, 5] and greater BMD [5, 6]. Limited studies have explored the independent effects of dynapenia and obesity on BMD or fracture risk. A recent systematic review [25] reported no difference in lumbar spine BMD (eight studies included; total *n* = 9014) among groups with obesity and sarcopenic obesity; the clinical significance of a statistically significant reduction in femoral neck BMD was unclear (six studies included; total *n* = 5608). It should be noted that heterogenous definitions of sarcopenic obesity (low lean mass, low muscle strength, and a combination of both) were included in this review. In addition, the risk of nonvertebral fracture was similar in people with sarcopenic or dynapenic obesity compared with people with or without obesity, suggesting no cumulative effect. However, these two studies and respective subgroups of people with sarcopenic obesity (*n* = 100‐128) were small [15, 26], and nonvertebral fractures have heterogeneous risk factors and mechanisms, especially in obesity (e.g., obesity increases ankle fracture risk but decreases hip fracture risk).

The aim of this study was to determine whether the fracture pattern observed in women living with obesity is modulated by dynapenia. In order to address this aim, we examined the independent effects of obesity and dynapenia on falls, BMD, and fracture risk. Fractures were divided into lower extremity fractures (obesity‐prone) and all other fractures (largely obesity‐protective). We hypothesized that dynapenia increased the risk of lower extremity fractures and counteracted the protective effects of obesity on all other fractures, and we speculated that this may relate to differences in either falls risk or BMD.

## METHODS

### Participants

A description of the UK Biobank has been published elsewhere [27]. Briefly, between 2006 and 2010, the UK Biobank recruited ~500,000 participants (5.5% response rate) aged 40 to 69 years from the general UK population [27]. Participants attended 1 of 22 assessment centers across the UK where they completed a touch‐screen questionnaire and an interview, underwent assessment of physical measures, and provided biological samples [27]. The results presented here are from the imaging visit, which began in 2014 and is ongoing. Recent comparison with population‐based studies suggests that risk factor associations in the UK Biobank are generalizable [28]. Participants who were male (owing to the low incidence of fractures; <3%); aged <60 years; had body mass index (BMI) < 18.5 kg/m^2^ (owing to the association between low BMI and fracture risk and the low number of participants within this group); or had missing data for BMI, waist circumference, handgrip strength (HGS), or the question on prior falls were excluded. The UK Biobank was approved by the North West Multicenter Research Ethics Committee, UK. Written informed consent was obtained prior to study entry. A Strengthening the Reporting of Observational Studies in Epidemiology (STROBE) checklist is included (Supporting Information Table S1) [29].

### Measures

#### Falls and fractures

Participants were asked whether they had any falls in the last year. Participants could respond that they had no falls, only one fall, or more than one fall. Participants with one or more falls were classified as “fallers,” and those with no falls or those who could not recall were classified as “non‐fallers.” Participants were asked whether they had fractured or broken any bones in the last 5 years. Participants were subsequently asked whether the fracture had resulted from a fall (i.e., from standing height). Participants who responded that the fracture had resulted from a fall were classified as “injurers.” Participants who responded that the fracture had not resulted from a fall or that they did not know were excluded from the fracture analysis. Participants were asked to report the fractured bone site or sites (i.e., spine, hip, wrist, leg, ankle, arm, or other bones). Based on previous literature [1, 2, 3, 4, 5], fracture types were classified as follows: 1) lower extremity (ankle, leg) fractures, sites that are “obesity‐prone;” and 2) other fractures (all other sites) or “obesity‐protective.” Participants with ankle or leg fractures were preferentially categorized into the lower extremity fracture group irrespective of whether other sites were fractured.

#### Covariates

Sociodemographic factors included age, self‐reported diabetes, alcohol status, and smoking status (prefer not to answer, never, previous, current). Individuals who preferred not to answer or did not know were assumed to never have smoked (*n* = 75), to never have had alcohol (*n* = 4), or to not have diabetes (*n* = 49). Weight was measured using a Tanita BC‐418 MA body composition analyzer (Tanita Europe, Amsterdam, the Netherlands) without shoes and heavy outer clothing. Height was measured using a Seca 202 height measure (Seca, Hamburg, Germany). BMI was calculated as weight (kilograms) divided by height (meters squared). Waist circumference was measured at the level of the umbilicus using a Wessex nonstretchable sprung tape. Maximal HGS was measured once on both right and left hands using a Jamar J00105 hydraulic hand dynamometer (Lafayette Instrument Co., Lafayette, Indiana); the maximal measure from either hand was used in this analysis. The UK Biobank protocol for data entry included computer‐generated warnings for implausible measurements. Dual‐energy x‐ray absorptiometry was performed using a GE‐Lunar iDXA (GE Healthcare, Madison, Wisconsin). Ethnicity was identified from the available baseline data of the UK Biobank because only 22% had this information at the imaging visit. Owing to large numbers, ethnicity was grouped as White (British, Irish, any other White background) or other ethnic group (prefer not to answer; Asian or Asian British; Chinese; other ethnic group; White and Black Caribbean; White and Black African; White and Asian; any other mixed background; Indian; Pakistani; any other Asian background; Caribbean; African; and those with missing data).

#### Dynapenia and obesity

Dynapenia was defined as the lowest tertile of HGS (≤21 kg). A tertile approach was chosen, similar to others [15, 16, 30], owing to a lack of consensus and to allow for an exploratory approach. Normal weight, overweight, and obesity were classified according to consensus definitions based on BMI [31].

### Statistical analysis

A χ^2^ test was used for comparison among categorical variables. Comparison among more than two groups was conducted using either one‐way ANOVA with Tukey post hoc test or Kruskal–Wallis test with Dunn post hoc test. Multiple logistic regression was used to examine the association among measures of obesity, dynapenia, or dynapenic obesity and self‐reported falls in the past 12 months, lower extremity fractures, and all other fractures in the past 5 years. Regression models were adjusted for age, measurement site, smoking status, self‐reported diabetes status, and alcohol status, with results expressed as odds ratios (ORs). Additional adjustment included right femoral neck BMD, HGS, or BMI. Physical activity (moderate activity minutes per day) was initially considered as a confounder for both falls and fractures, but adjustment did not alter results or conclusions, and it was deemed that the analysis was more impaired by missing or incomplete data for physical activity (*n* = 2791). Significance was accepted at *p* < 0.05. Analysis was undertaken using Stata version 16.1 (StataCorp LLC, College Station, Texas).

#### Individual variable models

Measures of obesity and dynapenia were explored separately as continuous variables. For continuous measures of dynapenia or obesity, *z* scores were calculated as an individual's result minus the population mean, divided by the population standard deviation.

#### Dynapenia by BMI category models

We included BMI categories divided by dynapenia status. There were six subgroups: 1) individuals with normal weight and no dynapenia; 2) individuals with dynapenia only; 3) individuals with overweight and without dynapenia; 4) individuals with overweight and dynapenia; 5) individuals with obesity only; and 6) individuals with dynapenic obesity. The group with normal weight without dynapenia was used as the reference group.

## RESULTS

### Baseline characteristics

Of the 17,175 women at the imaging visit aged 60 years or older, participants who did not answer the question about prior falls (*n* = 152); had a BMI < 18.5 kg/m^2^ (*n* = 206; 2 of whom experienced fractures from falls); or had incomplete measures for HGS (*n* = 617), BMI (*n* = 51), or waist circumference (*n* = 2) were excluded (Supporting Information Figure S1). A total of 16,147 women (aged 60‐82 years) were included and categorized according to dynapenia status (lowest tertile of HGS; <21 kg) and BMI categories (normal weight, overweight, or obesity; Table 1). BMI ranged from 18.5 to 69.6 kg/m^2^. Participants with dynapenia were older than their counterparts in the same BMI category (*p* < 0.001). BMI, waist circumference, and physical activity (minutes per day) were similar between participants with or without dynapenia in the same BMI category. Participants with dynapenic obesity (*p* < 0.05) and dynapenic overweight (*p* < 0.01) had lower BMD and *T* scores than counterparts in the same BMI category. Participants with normal weight and dynapenia had similar L1‐L4 BMD (*p* = 0.052) and *T* scores (*p* = 0.053) but lower femoral neck BMD and *T* scores (*p* < 0.001). However, differences were not clinically relevant (<5% or <0.5 *T* score) [32]. The proportion of fallers increased with both dynapenia and obesity status (*p* < 0.001).

**TABLE 1:** Characteristics of participants according to BMI category and dynapenia status

	Normal weight (BMI = 18.5‐24.9 kg/m^2^)		Obesity (BMI ≥ 30 kg/m^2^)		Overweight (BMI = 25‐29.9 kg/m^2^)	
	Normal strength,	Dynapenia,	Normal strength,	Dynapenia,	Normal strength,	Dynapenia,
	*n* = 5279 (32.7%)	*n* = 2252 (14%)	*n* = 1812 (11.2%)	*n* = 985 (6.1%)	*n* = 3941 (24.4%)	*n* = 1878 (11.6%)
Age (y), median (IQR)	67 (7)	69 (8)	66 (7)	68 (8)	67 (8)	69 (8)
BMI (kg/m^2^), median (IQR)	22.7 (2.5)	22.8 (2.5)	32.9 (4.2)	32.6 (4.1)	27.0 (2.3)	27.1 (2.5)
Waist circumference (cm), median (IQR)	75 (10)	75 (9)	99 (12)	99 (12)	86 (10)	86 (10)
HGS (kg), median (IQR)	26 (6)	18 (4)	26 (5)	18 (4)	26 (5)	18 (4)
Ethnicity, *n* (%)						
White	5191 (98)	2182 (97)	1751 (97)	961 (97)	3857 (98)	1823 (97)
Other ethnic group	88 (2)	70 (2)	61 (3)	24 (3)	84 (2)	56 (3)
BMD^a^, median (IQR)						
L1‐L4 BMD (g/cm^2^)	1.04 (0.21)	1.02 (0.21)	1.19 (0.25)	1.16 (0.25)	1.11 (0.23)	1.09 (0.23)
L1‐L4 *T* score	−1.19 (1.76)	−1.31 (1.78)	0.04 (2.05)	−0.15 (2.10)	−0.56 (1.89)	−0.72 (1.93)
RFN BMD (g/cm^2^)	0.83 (0.14)	0.81 (0.14)	0.93 (0.17)	0.90 (0.16)	0.88 (0.16)	0.86 (0.15)
RFN *T* score	−1.27 (1.19)	−1.42 (1.21)	−0.44 (1.43)	−0.71 (1.37)	−0.88 (1.31)	−1.03 (1.28)
Physical activity level, median (IQR)						
Mod. activity (min/d)^b^	60 (60)	60 (60)	40 (40)	45 (40)	60 (50)	60 (60)
Mod. activity (d/wk)^c^	5 (4)	5 (4)	4 (4)	3 (3)	4 (3)	4 (5)
Falls, *n* (%)	1051 (19.91)	576 (25.58)	465 (25.66)	294 (29.85)	887 (22.51)	520 (27.69)
More than one fall, *n* (%)	253 (4.79)	167 (7.41)	141 (7.78)	86 (8.73)	238 (6.04)	177 (9.42)
All fractures, *n*	470	291	152	114	361	184
Lower extremity, *n*	58	47	42	31	85	41
Other fractures, *n*	412	244	110	83	276	143
Diabetes, *n* (%)	77 (1.46)	48 (2.13)	185 (10.21)	119 (12.08)	150 (3.81)	111 (5.91)
Smokers, *n* (%)						
Never	3462 (65.58)	1523 (67.63)	1091 (60.21)	619 (62.84)	2484 (63.03)	1218 (64.86)
Previous	1707 (32.34)	669 (29.71)	683 (36.69)	344 (34.92)	1361 (34.53)	626 (33.33)
Current	110 (2.08)	60 (2.66)	38 (2.10)	22 (2.23)	96 (2.44)	34 (1.81)
Alcohol, *n* (%)						
Never	181 (3.43)	125 (5.55)	103 (5.68)	67 (6.80)	153 (3.88)	121 (6.44)
Previous	159 (3.01)	80 (3.55)	73 (4.03)	63 (6.40)	132 (3.35)	84 (4.47)
Current	4939 (93.56)	2047 (90.90)	1636 (90.29)	855 (86.80)	3656 (92.77)	1673 (89.08)

Abbreviations: BMD, bone mineral density; HGS, handgrip strength; Mod., moderate; RFN, right femoral neck.

^a^ Missing measurements for L1‐L4 BMD: *n* = 3439; L1‐L4 *T* score: *n* = 3445; RFN BMD: *n* = 3271; and RFN *T* score: *n* = 3278.

^b^ A total of 1334 participants did not know/answer this question, and 2357 participants had missing measurements.

^c^ 578 participants did not know/answer this question.

### Falls and fractures

**FIGURE 1:**
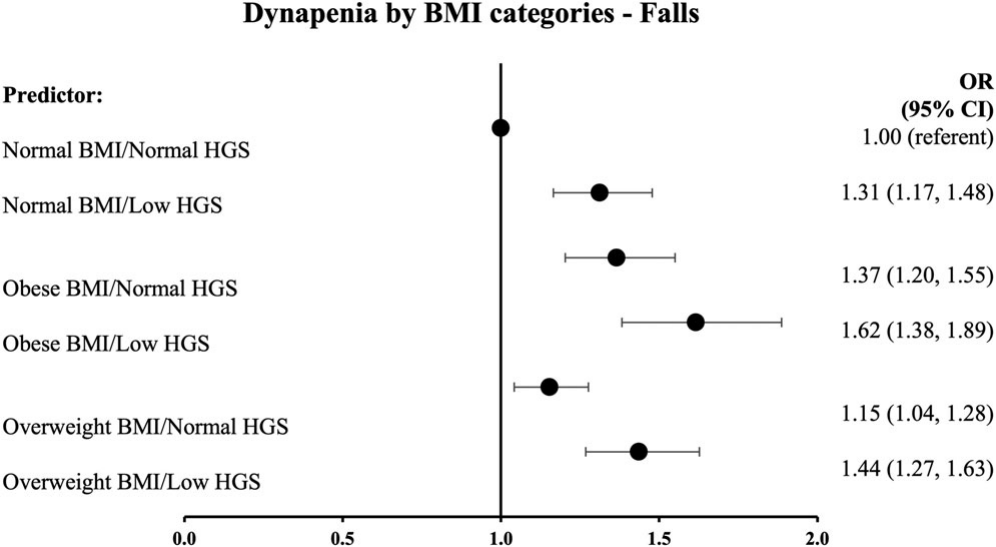
Association between dynapenia by BMI categories and falls risk. Adjusted for age, measurement center, smoking status, self‐reported “diabetes” status and alcohol status. *p > 0.05. HGS, handgrip strength; OR, odds ratio

A total of 3793 (23.5%) women fell, of whom 1062 reported more than one fall. Across all BMI categories, dynapenia appeared to increase the risk of falling (Figure 1). In the past 5 years, 577 women reported a fracture from causes other than a fall and were excluded from the fracture analysis. Therefore, 1413 of 15,570 (9.1%) eligible women reported a fracture due to a fall. The proportion of injurers according to BMI and HGS tertile is presented in Table 2. Injurers were classified as lower extremity (*n* = 295; 1.9%) or other fractures (*n* = 1118; 7.2%). Of the 295 lower extremity injurers, 58 (19.6%) women also reported other fractures (spine, arm, wrist, other bones), and 9 (3%) reported both ankle and leg fractures. In total, 1572 fractures were recorded. The most frequently reported fractures were “other bones” (577; 37%), followed by wrist (461; 29%) and ankle (230; 15%).

**TABLE 2:** Proportion of participants classified by both tertiles of HGS and BMI who were identified as being in the all other fractures or lower extremity fractures groups

	All other fractures		
HGS tertile	Normal weight, *n* (%)	Obesity, *n* (%)	Overweight, *n* (%)
High (≥26 kg)	191 (6.95)	45 (4.74)	142 (6.70)
Medium (21‐25.9 kg)	178 (7.68)	55 (6.77)	106 (6.22)
Low (0‐20.9 kg)	202 (9.41)	75 (7.81)	124 (6.84)

	Lower extremity fractures		
HGS tertile	Normal weight, *n* (%)	Obesity, *n* (%)	Overweight, *n* (%)
High (≥26 kg)	27 (0.98)	25 (2.63)	39 (1.84)
Medium (21‐25.9 kg)	31 (1.13)	17 (2.09)	44 (2.58)
Low (0‐20.9 kg)	41 (1.49)	30 (3.13)	41 (2.26)

Abbreviations: HGS, handgrip strength.

### Association between falls, fractures, and measures of muscle strength and obesity

The association between measures of obesity and dynapenia as continuous variables and falls, lower extremity fractures, and all other fracture risk are shown in Table 3. In a multivariable model, obesity, as determined either by greater BMI (OR 1.12; 95% confidence interval [CI]: 1.08‐1.16) or waist circumference (OR 1.14; 95% CI: 1.09‐1.18), and lower HGS (OR 0.86; 95% CI: 0.83‐0.90) had independent effects on falls risk.

**TABLE 3:** Associations, expressed as ORs, among *z* scores of BMI, waist circumference, HGS, and falls (lower extremity fractures and all other fractures)

	Falls			Lower extremity fractures			All other fractures		
	OR	95% CI	*p*	OR	95% CI	*p*	OR	95% CI	*p*
Model 1									
BMI	1.12	1.08‐1.16	**0.000**	1.25	1.13‐1.39	**0.000**	0.91	0.85‐0.97	**0.005**
Waist circumference	1.14	1.10‐1.18	**0.000**	1.27	1.14‐1.42	**0.000**	0.96	0.90‐1.02	0.190
HGS	0.86	0.83‐0.89	**0.000**	0.86	0.76‐0.97	**0.015**	0.90	0.84‐0.96	**0.002**
Model 1 + HGS z scores									
BMI	1.12	1.08‐1.16	**0.000**	1.25	1.12‐1.38	**0.000**	0.91	0.85‐0.97	**0.004**
Waist circumference	1.14	1.09‐1.18	**0.000**	1.27	1.13‐1.41	**0.000**	0.96	0.90‐1.02	0.168
HGS^a^	0.86	0.83‐0.90	**0.000**	0.87	0.77‐0.98	**0.023**	0.90	0.84‐0.96	**0.001**
Model 1 + RFN BMD									
BMI	‐	‐	‐	1.30	1.15‐1.46	**0.000**	0.98	0.91‐1.06	0.584
Waist circumference	‐	‐	‐	1.33	1.17‐1.52	**0.000**	1.02	0.95‐1.10	0.601
HGS	‐	‐	‐	0.84	0.74‐0.97	**0.014**	0.92	0.86‐0.99	**0.033**

*Note*: *Z* score for HGS = 5.71 kg; BMI = 4.56 kg/m^2^; and waist circumference = 11.57 cm. Model 1: adjusted for age, measurement center, diabetes status, smoking status, and alcohol status.

Abbreviations: BMD, bone mineral density; HGS, handgrip strength; OR, odds ratio; RFN, right femoral neck.

*p* < 0.05 are in bold.

^a^ HGS = Model 1 + BMI *z* scores.

Obesity or dynapenia as continuous variables, irrespective of BMD, were associated with a greater risk of lower extremity fractures, with evidence of an independent effect of both obesity and dynapenia (Table 3). In contrast, obesity was protective of all other fractures, whereas low HGS appeared to be associated with a greater risk. These findings remained when analyzed according to BMI category, with an increased risk of lower extremity fracture and lower risk of other fractures in people with overweight or obesity (Supporting Information Tables S2 and S3). Mediation analyses showed that BMI and HGS as *z* scores partially mediate each other (direct and indirect effects *p* < 0.05) in their associations with both lower extremity or other fracture risk.

### Association between fractures and dynapenia according to BMI category

**FIGURE 2:**
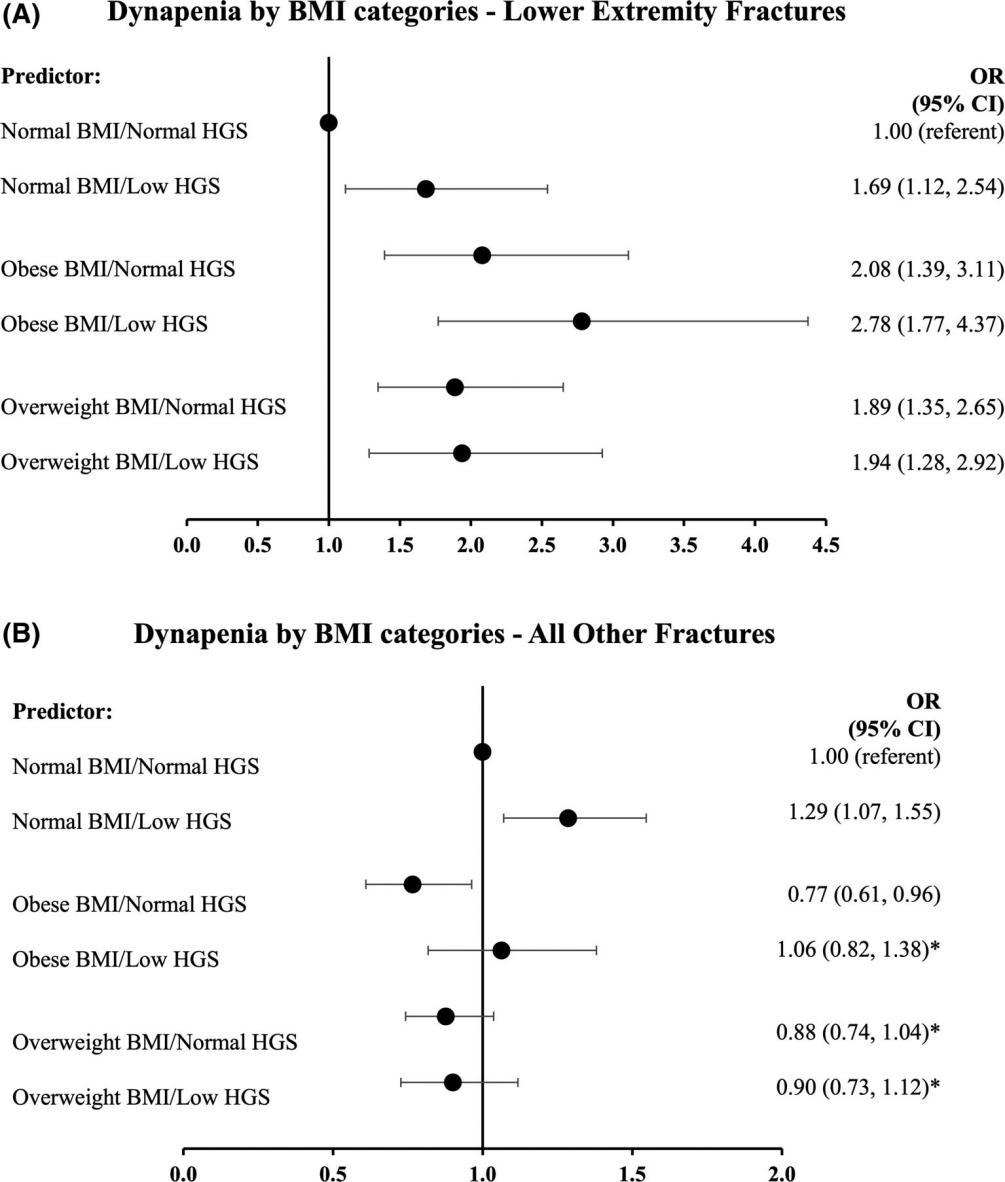
Association among (**A**) lower extremity or (**B**) all other fractures by dynapenia and obesity status. Adjusted for age, measurement center, smoking status, self‐reported “diabetes” status, and alcohol status. *p > 0.05. HGS, handgrip strength; OR, odds ratio

Compared with the group with normal weight, overweight and obesity were associated with a greater risk of lower extremity fractures (Figure 2A), with the greatest risk estimate seen in people with obesity (OR 2.08; 95% CI: 1.39‐3.11) or with dynapenic obesity (OR 2.78; 95% CI: 1.77‐4.37). The risk of lower extremity fracture among individuals with both normal weight and dynapenia was also greater than those with normal weight alone (OR 1.69; 95% CI: 1.12‐2.54). These findings support our continuous analysis whereby obesity and dynapenia appear to have individual and independent negative associations with lower extremity fracture.

Findings from the categorical analysis of BMI and dynapenia (Figure 2B) confirm our continuous analysis (Table 3) that BMI within the obesity category was protective of all other fractures (OR 0.77; 95% CI: 0.61‐0.96), but this effect was negated by the presence of low HGS in which fracture risk was similar to that of the group with normal weight (OR 1.06; 95% CI: 0.82‐1.38). Participants who had overweight with (OR 0.90; 95% CI: 0.73‐1.12) or without dynapenia (OR 0.88; 95% CI: 0.74‐1.04) had a similar risk of all other fractures to women with normal weight. However, individuals with normal BMI and dynapenia had a greater risk of all other fractures (OR 1.29; 95% CI: 1.07‐1.55) than those who had normal weight with normal strength. Owing to the large BMI range within this study, additional categorical analysis excluding participants with BMI ≥ 40 kg/m^2^ (*n* = 188) found similar findings for both lower extremity and other fractures (Supporting Information Figure S2).

## DISCUSSION

In this large, cross‐sectional, retrospective study of women (aged 60‐82 years), we have demonstrated the independent effects of obesity and dynapenia on risk of falls. We have shown that dynapenia confers an increased risk of all types of fractures. In contrast, the effects of obesity on fracture risk are site‐specific. Lower extremity fracture risk is increased with both obesity (whether measured using BMI or waist circumference) and dynapenia, irrespective of BMD. For all other fractures (wrist, arm, spine, hip, other bones), obesity is associated with a reduced risk of fracture, except when accompanied by dynapenia.

A recent systematic review has shown that individuals with “sarcopenic obesity” (obesity with reduced muscle mass or strength) have a similar risk of nonvertebral fractures to individuals with obesity alone [25], thereby suggesting that there is no cumulative effect of sarcopenia on fracture risk. However, the available studies were relatively small, containing small subgroups [15, 26] and failing to consider the site‐specific nature of fractures in people with obesity. Our findings are particularly novel because we have used a large cohort and recognized the site‐specific fracture risk in people living with obesity. Our results reinforce the evidence regarding the site‐specific nature of fractures in people with obesity, with their greater risk of sustaining ankle or leg fractures [1, 2, 3] but a reduced risk of other fractures [2, 4, 5]. However, our findings of the interaction between obesity and dynapenia on fracture risk are novel, namely that obesity increases the risk of lower extremity fractures (ankle, leg) and that dynapenia negates the positive effect of obesity on other fracture risk (wrist, arm, hip, spine, other bones). Consideration of both body weight and muscle strength are clearly relevant for determining and stratifying fall and fracture risk.

It is unclear the mechanism by which dynapenia may negate the protective effect of obesity on risk of other fractures. BMD is a well‐acknowledged risk factor for fracture [32]; however, measurement of BMD is problematic in people with obesity because of confounding by body mass and bone size [33]. However, other quantitative computed tomography methods also support higher BMD, denser cortices, and increased trabecular thickness and number in people with obesity [7]. Comparison was made between groups with obesity and dynapenic obesity who would both be subject to the same measurement artifact, and, similar to previous studies [34, 35], BMD was reduced in the group with dynapenic obesity, although the difference was small and unlikely to translate into a clinically significant difference in fracture risk [32]. The pathophysiology of dynapenia is multifactorial and can result from malnutrition, reduced physical activity, or comorbid disease [19]; therefore, it is possible that these factors may also contribute to the negating effect of dynapenia, although we were unable to consider these in our analysis. Our findings tentatively suggest that the greater risk of fracture may relate to the cumulative falls risk observed, bearing in mind the differing recall period of both outcomes in the UK Biobank.

The majority of fractures result from falls from a standing height [36]. Separately, obesity and dynapenia are associated with a greater risk of falls [20, 21, 22], with growing evidence of a cumulative effect on falls risk, greater than from either phenotype alone [17, 18, 23]. Our findings support the notion of a greater impact or more injurious fall type in people with obesity [37]. Biomechanically, abdominal obesity is associated with an anterior shift in the center of mass requiring greater torque for stabilization [38]. Moreover, reduced soft tissue padding at the lower extremities, in combination with a high impact fall, may render these sites more liable to fracture in obesity [1]. Our findings are similar to those of Nielson et al. [39] who demonstrated that obesity was associated with greater risk of lower extremity fracture in 5995 older men (mean follow‐up of 7 years), but, in contrast with our results, the effect of obesity was attenuated by reduced physical performance and prior fracture history. Objective measures of lower limb performance were not available in the UK Biobank, and, as such, it requires further consideration as to whether other physical performance measures, especially of the lower extremities, may better relate to lower extremity fractures in women.

Our findings have important clinical implications suggesting that lifestyle interventions that target low muscle strength (either preventing or improving dynapenia), either alone or with measures to reduce excess body weight, could reduce both the risk of falls and fractures in women. Although evidence in younger adults suggests that modest weight reduction achieves greater benefits in balance [40, 41], findings in older adults with obesity have shown that weight loss alone exacerbates dynapenia [42]. Furthermore, weight loss without concomitant resistance or weight‐bearing exercise can result in loss of BMD, particularly around the hip [43, 44], thereby potentially counteracting the reduction in fracture risk derived from a lesser falls risk. Notwithstanding, achieving a healthy weight is a public health priority considering the association of obesity with multiple chronic conditions/comorbidities (e.g., type 2 diabetes, cardiovascular disease, cancer) [31] in addition to falls and fractures risk. Indeed, the greatest gains in physical performance were derived from combined dietary and physical activity interventions [45], further highlighting the relevance of increasing physical activity and functional measurements, particularly of muscle strength, in weight management programs.

The limitations of this study must be acknowledged. First, we were unable to validate the occurrence of fractures because International Classification of Diseases codes were only available for those who were admitted to hospital. Therefore, it is possible that participants may have misclassified or underreported (e.g., spine) their (self‐reported) fracture sites. Moreover, individuals with lower extremity fractures also had other fractures; therefore, this may have affected associations. Second, this was a retrospective study, and the inferences drawn should be viewed with caution because dynapenia or obesity may have occurred secondary to a fall or a fracture rather than being a driving pathophysiological factor. Longitudinal analysis was not feasible owing to either an inadequate sample size at both visits or variability in baseline measurement collection time (5‐14 years) prior to the outcome (previous 5 years). Third, HGS is a surrogate measure of lower limb strength [13] but can be affected by other factors such as nutrition. Next, we could not replicate this present analysis in male individuals because of the low prevalence of fractures in this group; therefore, further work is required to confirm these findings in male individuals. Finally, the limitations of the UK Biobank in relation to generalizability must be considered, which include its low response rate, limited ethnic diversity, lower overall prevalence of overweight and obesity than the UK population, and potential healthy volunteer bias (e.g., lower rates of diabetes, narrow socioeconomic backgrounds). However, strengths of the UK Biobank include its significant sample size and the detailed phenotyping available, with evidence supporting the notion that risk factor associations may be generalizable [28].

We have demonstrated that obesity and dynapenia have independent effects on falls risk, but the relationship among obesity, dynapenia, and fracture risk is anatomically site‐specific. Dynapenia negates the protective effects of obesity on fractures of the wrist, arm, hip, spine, and other bones, suggesting that greater falls risk or other risk factors, rather than differences in BMD, may explain the negative effect of dynapenia. In contrast, both obesity and dynapenia are associated with a greater risk of lower extremity fractures, independently of BMD, suggesting fall type or fall force may be a mediating factor. These findings require validation in prospective analysis and in men but they may have important clinical implications relating to risk identification and prevention of falls and fractures in a growing population living with overweight and obesity.

## FUNDING INFORMATION

This work was supported by a studentship from the Medical Research Council (MRC) and Versus Arthritis as part of the MRC Versus Arthritis Centre for Integrated Research into Musculoskeletal Ageing (CIMA; MR/R502182/1). The MRC Versus Arthritis Centre for Integrated Research into Musculoskeletal is a collaboration among the universities of Liverpool, Sheffield, and Newcastle.

## CONFLICT OF INTEREST

The authors declared no conflict of interest.

## Supporting information


**FIGURE S1**Flow diagram of number of individuals at each stage examined for eligibility.

**FIGURE S2** Association among (**A**) lower extremity or (**B**) all other fractures by dynapenia and obesity status, excluding those with BMI ≥ 40 kg/m^2^ (*n* = 188). Adjusted for age, measurement center, smoking status, self‐reported “diabetes” status, and alcohol status. *p > 0.05. Abbreviations: BMI, body mass index; HGS, handgrip strength.

**TABLE S1**STROBE statement: checklist of items that should be included in reports of cohort studies

**TABLE S2**Association among lower extremity fractures and obesity

**TABLE S3** Association among all other fractures and obesity
