# Supplementary material for: Reduced muscle strength (dynapenia) in women with obesity confers a greater risk of falls and fractures in the UK Biobank
Source: Obesity (Silver Spring). 2022 Dec 11;31(2):496–505. doi: 10.1002/oby.23609 (PMC10108064; doi:10.1002/oby.23609)
Supplement: Supplementary file 1 — FIGURE S1Flow diagram of number of individuals at each stage examined for eligibility. [file OBY-31-496-s001.pdf]

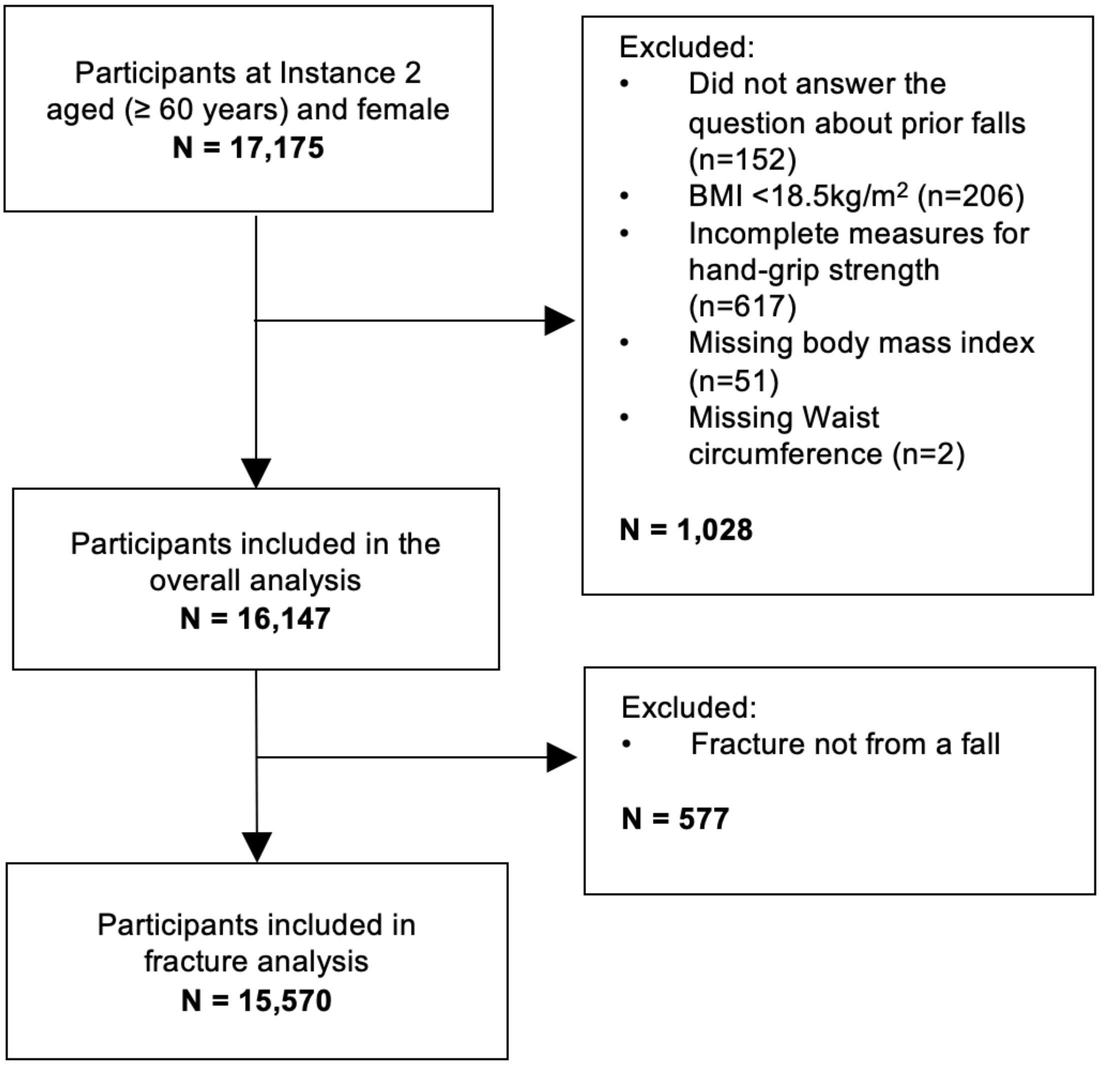

Supplementary Figure 1: Flow diagram of number of individuals at each stage examined for eligibility.
