# Supplementary material for: Reduced muscle strength (dynapenia) in women with obesity confers a greater risk of falls and fractures in the UK Biobank
Source: Obesity (Silver Spring). 2022 Dec 11;31(2):496–505. doi: 10.1002/oby.23609 (PMC10108064; doi:10.1002/oby.23609)
Supplement: Supplementary file 2 — FIGURE S2 Association among (A) lower extremity or (B) all other fractures by dynapenia and obesity status, excluding those with BMI ≥ 40 kg/m2 (n = 188). Adjusted for age, measurement center, smoking status, self‐reported “diabetes” status, and alcohol status. *p > 0.05. Abbreviations: BMI, body mass index; HGS, handgrip strength. [file OBY-31-496-s005.pdf]

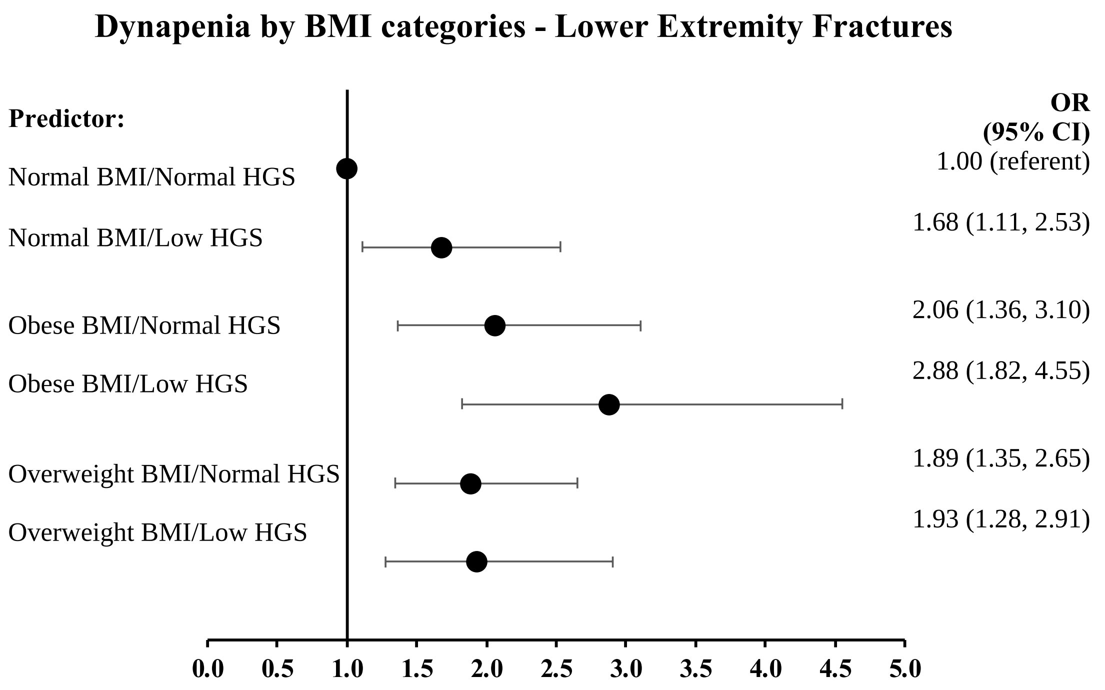

(a) Lower extremity fractures (ankle, leg)

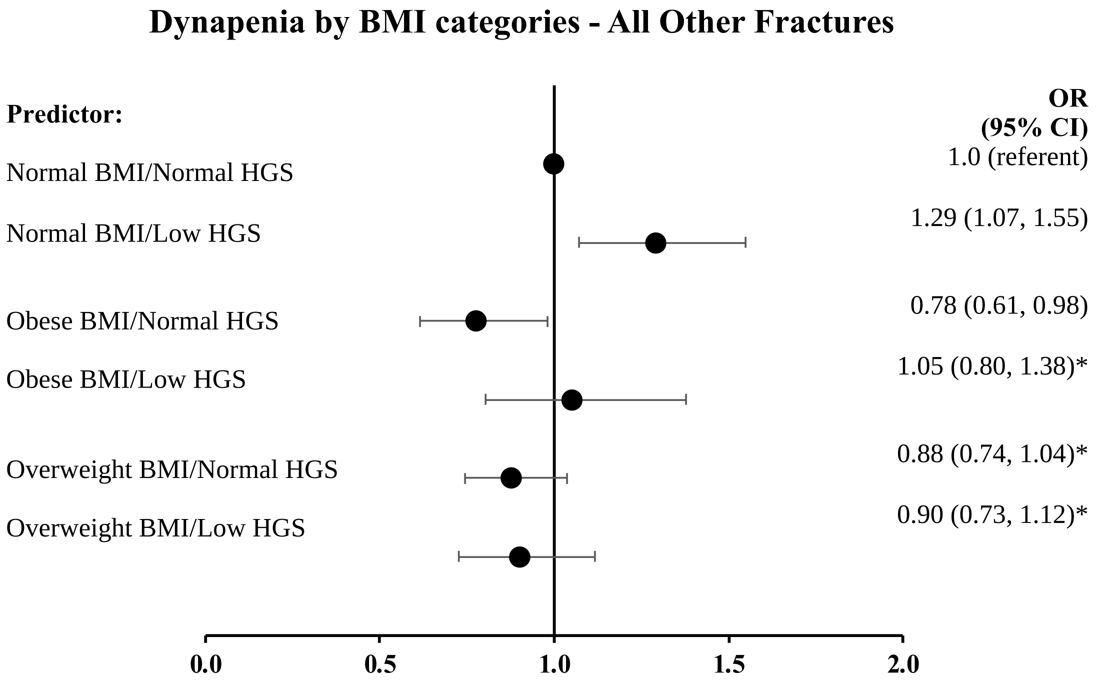

(b) All other fractures (hip, spine, wrist, arm, other bones)

Supplementary Figure 2 Association between (a) lower extremity or (b) all other fractures by dynapenia and obesity status excluding those with BMI ≥ 40kg/m^2 (n = 188)

Adjusted for age, measurement centre, smoking status, self-reported 'diabetes' status, alcohol status. * p > 0.05. Abbreviations: BMI = Body Mass Index; HGS = Hand Grip Strength.
