# Supplementary material for: Reduced muscle strength (dynapenia) in women with obesity confers a greater risk of falls and fractures in the UK Biobank
Source: Obesity (Silver Spring). 2022 Dec 11;31(2):496–505. doi: 10.1002/oby.23609 (PMC10108064; doi:10.1002/oby.23609)
Supplement: Supplementary file 4 — TABLE S2Association among lower extremity fractures and obesity [file OBY-31-496-s002.docx]

## Table S2 Association between lower extremity fractures and obesity

|  | Normal Weight | Overweight | | | | Obese | | | |
| --- | --- | --- | --- | --- | --- | --- | --- | --- | --- |
| BMI Categories | **Ref** | **OR** | **95% CI** | | **P** | **OR** | **95% CI** | | **P** |
| Model 1 | 1 | 1.57 | 1.20 | 2.06 | **0.001** | 1.90 | 1.39 | 2.60 | **0.000** |
| Model 1 + RFN BMD | 1 | 1.55 | 1.14 | 2.10 | **0.005** | 2.03 | 1.43 | 2.91 | **0.000** |
| Model 1 + dynapenia (HGS) | 1 | 1.57 | 1.20 | 2.06 | **0.001** | 1.88 | 1.38 | 2.57 | **0.000** |
| Waist Categories |  |  | **Medium Risk** | |  |  | **High Risk** | |  |
| Model 1 | 1 | 1.38 | 1.03 | 1.85 | **0.033** | 1.69 | 1.27 | 2.25 | **0.000** |
| Model 1 + RFN BMD | 1 | 1.39 | 1.00 | 1.93 | 0.053 | 1.83 | 1.32 | 2.54 | **0.000** |
| Model 1 + dynapenia (HGS) | 1 | 1.37 | 1.02 | 1.84 | **0.037** | 1.67 | 1.25 | 2.22 | **0.000** |

Model 1 - Adjusted for age, measurement centre, smoking status, self-reported ‘diabetes’ status, alcohol status. Abbreviations: BMI = Body Mass Index; HGS = Hand Grip Strength; RFN BMD = right femoral neck bone mineral density. Waist categories refer to normal (<80cm), medium risk (80-88cm), high risk (>88cm) (31).
