# Supplementary material for: Reduced muscle strength (dynapenia) in women with obesity confers a greater risk of falls and fractures in the UK Biobank
Source: Obesity (Silver Spring). 2022 Dec 11;31(2):496–505. doi: 10.1002/oby.23609 (PMC10108064; doi:10.1002/oby.23609)
Supplement: Supplementary file 5 — TABLE S3 Association among all other fractures and obesity [file OBY-31-496-s003.docx]

## Table S3 Association between all other fractures and obesity

|  | Normal | Overweight | | | | Obese | | | |
| --- | --- | --- | --- | --- | --- | --- | --- | --- | --- |
| BMI Categories | **Ref** | **OR** | **95% CI** | | **P** | **OR** | **95% CI** | | **P** |
| Model 1 | 1 | 0.81 | 0.71 | 0.93 | **0.003** | 0.79 | 0.66 | 0.95 | **0.011** |
| Model 1 + RFN BMD | 1 | 0.86 | 0.73 | 1.00 | 0.058 | 0.96 | 0.78 | 1.18 | 0.685 |
| Model 1 + dynapenia (HGS) | 1 | 0.81 | 0.71 | 0.93 | **0.002** | 0.78 | 0.66 | 0.94 | **0.008** |
| Waist Categories |  |  | **Medium Risk** | |  |  | **High Risk** | |  |
| Model 1 | 1 | 0.91 | 0.78 | 1.05 | 0.201 | 0.92 | 0.79 | 1.07 | 0.275 |
| Model 1 + RFN BMD | 1 | 0.99 | 0.83 | 1.17 | 0.867 | 1.05 | 0.88 | 1.25 | 0.609 |
| Model 1 + dynapenia (HGS) | 1 | 0.90 | 0.78 | 1.05 | 0.180 | 0.91 | 0.78 | 1.06 | 0.232 |

Model 1 - Adjusted for age, measurement centre, smoking status, self-reported ‘diabetes’ status, alcohol status. Abbreviations: BMI = Body Mass Index; HGS = Hand Grip Strength. Waist categories refer to normal (<80cm), medium risk (80-88cm), high risk (>88cm) (31).
